# Some Considerations on the Operation for Cleft Palate

**Published:** 1906-02

**Authors:** 


### Some Considerations on the Operation for Cleft Palate.

From the wide prevalence of congenital harelip and cleft palate among all branches of the human family, and from its occurrence even in many of the lower animals, we are justified in thinking that this arrest of the development of the face must have occurred from the time of the first appearance of man on the earth. Yet, strange to say, we are able to find only scanty references to the deformities until nearly modern times. Speculations as to the etiology of this condition have been long rife and the popular mind has almost universally agreed that "maternal impressions" are responsible for most of the instances of its occurrence. Vivid dreams, frights, and woundings are all credited with the production of this maldevelopment and though medicine for the most part has discarded these explanations, we must confess that modern science has no satisfactory reasons to offer in their stead. We can not doubt that surgeons have seen and examined cases of harelip and cleft palate from the time when surgery first arose, but we can find no trace of any attempt having been made to rectify the abnormality until the seventeenth century, and the treatment then was limited to closing the cleft in the lip. In these cheiloplastic operations, however, the progress of knowledge and the widening of experience have led to results which are all that can be wished, and it may be said that at the present time all surgeons are agreed as to the main principles governing the operation of closing a harelip, and hardly any one can be found to advocate the postponement of the operation beyond the second year of life.

The unanimity which exists as to the method of treatment of harelip is not to be found amongst surgeons in the treatment of congenital cleft of the palate. It was long after the first attempts at operations on harelip before a surgeon was found daring enough to essay the closure of a cleft palate, and for a long time these attempts were confined to clefts of the velum palati in which the separation of the two halves was but slight. The credit of establishing the operation for cleft of the soft palate must be given to Roux. For a long time it was considered advisable to postpone this operation until the patient was of an age to appreciate the importance of what was done and sufficiently intelligent to co-operate with the surgeon not only in the operation itself but also in the after-treatment. This was the course pursued by the surgeon as a routine not only in the time before the introduction of anesthetics, but after, for even when ether or chloroform was used for all other operations it was considered advisable to forego the advantages of anesthesia in operations of the patient and partly from a fear that asphyxia might result from some of the blood (and the hemorrhage is often profuse) finding its way into the air passages. Closure of clefts of the hard palate was not attempted for many years after staphylorrhaphy had become a frequent operation. Warren, of Boston, was the introducer of this advance, which took place in 1843; he raised the mucoperiosteum from the palate bones on each side and brought them together in the middle line. In 1869 the next great step of progress was made. Sir Thomas Smith showed that the risk of asphyxia from hemorrhage had been much exaggerated if reasonable care were taken, and that therefore it was justifiable to administer anesthetics for the operation. By means of a cleverly contrived gag Sir Thomas Smith was able to fix the jaws and tongue so that the willing and intelligent assistance of the patient was not required. The administration of an anesthetic in such cases at once became a great boon to both patient and surgeon, and since that time some form of anesthetic has always been employed in the operation of closing a cleft of either the hard or the soft palate.

There has been on the whole a tendency towards the performance of these operations at an earlier age than formerly, but the question of the best age at which staphylorrhaphy and uranoplasty should be done is now the most keenly discussed of any connected with the subject, and recently the matter has received attention in many directions. Mr. W. Arbuthnot Lane has published a work on cleft palate and harelip in which he has given a *résumé* of many papers which he has written at various times.[1] Mr. Lane is the chief advocate of the very early performance of the operation for harelip and cleft palate. The arguments in favor of an early operation are these: the abnormality is an arrest in development, and,

---

[1]Cleft Palate and Harelip, London Medical Publishing Company, 1905.

therefore, the sooner the parts are placed in their natural positions the more likely is development to proceed along normal lines. Moreover, if the palate is reconstituted before any attempts at speaking are made, it is highly probable that the voice will possess none of those qualities which are connoted by the word "nasal." The majority of surgeons, however, both in this country and elsewhere, are opposed to very early operations, and they advance in support of their opinions the following arguments: the risk of sepsis at a very early age is greater, the tissues are more liable to slough and are more lacerable than later, and they say that the older children are more easily kept quiet after the operation and that the voice differs but little from the normal if the cleft is closed before the fourth or fifth year. Who can decide? From a theoretical point of view no one, we think, will venture to deny that the earlier operation would be desirable, as rendering it possible for the child to develop along normal lines, provided always that the risks of the operation itself are equal to those obtained in older children. But these are exactly the two main propositions put forward by Mr. Lane which are denied by those who oppose his method. The surgeons who favor some delay in undertaking these operations say, as we have already mentioned, that at the early age advised by Mr. Lane—that is, within the first six months of life—the tissues are very lacerable and slough readily and that the risk to life is by no means small, while with the later operation the results are quite as good, if not better, and there is no real danger to life. We do not take upon ourselves definitely to decide who is right in this important matter; indeed, time alone can show which view is the sound one, but we are inclined to think that the origin of the differences of opinion is mainly to be found in the tendencies of the operation. From the first inception of staphylorrhaphy to the present time the well-marked tendency has been to attempt the closure of these maldevelopments at an earlier age than before. It may well be that the ideal age is not necessarily to be found in the first few months of life; yet when we consider that the sooner the cleft is closed the more readily will development approach to the development of a normal child, we can not deny that the argument in favor of the early operation is very strong. The danger of sepsis, of sloughing of the flap, and of impairment of the general health of the child can not be ignored, but the improvements of modern asepsic processes will go far to counteract any depressing influence of the operation. The individual skill of the surgeon is also a factor which can not be ignored. Bearing in mind all the considerations we can not but feel that if due care be exercised the early operation need give rise to little increase of danger. This being so, the advantages of a restoration of the continuity of the parts before any attempts at speech are made are very great and go far to outweigh the possible slight increase of risk. — *London Lancet.*

